# Gefitinib treatment after exfoliative dermatitis induced by sequential aumolertinib and furmonertinib in lung adenocarcinoma: a case report

**DOI:** 10.3389/fonc.2026.1843848

**Published:** 2026-07-15

**Authors:** Weifen Xiang, Xiaoyan Hu, Juan Wei, Zhen Cheng, Xiaofang Dong

**Affiliations:** Department of Oncology, Dongyang People’s Hospital Affiliated to Wenzhou Medical University, Dongyang, Zhejiang, China

**Keywords:** adverse drug reaction, aumolertinib, exfoliative dermatitis, furmonertinib, gefitinib, lung adenocarcinoma

## Abstract

This case report describes a 74-year-old female patient with lung adenocarcinoma harboring an *EGFR* exon 21 L858R variant and brain metastases. During sequential treatment with two third-generation epidermal growth factor receptor–tyrosine kinase inhibitors (EGFR-TKIs), aumolertinib (formerly known as almonertinib) and furmonertinib, the patient developed severe exfoliative dermatitis (ED). Given the patient’s poor performance status, chemotherapy was declined, and treatment was subsequently switched to the first-generation EGFR-TKI gefitinib. Gefitinib was well tolerated, and no recurrence of cutaneous reactions was observed. Follow-up chest computed tomography demonstrated stable disease, with disease control maintained for more than four months. This case highlights the importance of careful monitoring for severe and potentially life-threatening cutaneous adverse reactions, including ED, during treatment with third-generation EGFR-TKIs. The clinical course also suggests differences in cutaneous toxicity profiles among EGFR-TKIs of different generations. For patients who develop severe cutaneous toxicity with third-generation EGFR-TKIs, switching to a first-generation EGFR-TKI may represent a safe, feasible, and effective therapeutic alternative.

## 1 Introduction

Exfoliative dermatitis (ED) is a severe inflammatory skin disorder characterized by diffuse erythema and desquamation involving more than 90% of the body surface area (1), with potentially life-threatening complications including hemodynamic instability, metabolic disturbances, and infection (2, 3). The condition may develop acutely within hours or progress chronically over several days to weeks and is typically accompanied by generalized pruritus and systemic symptoms such as fever, chills, and nausea (4). Approximately 50% of patients present with lymphadenopathy, splenomegaly, or hepatomegaly (5). ED has a complex etiology, encompassing congenital, infectious, inflammatory, immune-mediated, neoplastic, iatrogenic, and idiopathic factors, with drug hypersensitivity representing the second most common cause (5, 6). Various medications, including antineoplastic agents, antibiotics, and other medications have been associated with the development of ED (7). The underlying immunopathological mechanisms are not fully understood but may involve delayed hypersensitivity reactions, anaphylactoid reactions, and antigen–antibody interactions, with the specific mechanism influenced by both the causative agent and host-related factors (8). The onset of ED may occur from hours to months after drug exposure, and the clinical course may be prolonged. Epidermal growth factor receptor–tyrosine kinase inhibitors (EGFR-TKIs) are established first-line therapies for advanced non-small cell lung cancer (NSCLC) harboring *EGFR* variants. Cutaneous toxicity is relatively common but is generally mild, whereas severe reactions such as ED are rare. Compared with first-generation EGFR-TKIs, third-generation EGFR-TKIs are associated with a lower incidence of skin-related toxic reactions (9). Life-threatening severe cutaneous adverse reactions, such as ED, are rare. The incidence of ED has not been clearly established in large-scale clinical studies, and most of the available clinical literature consists of case reports. The present case report describes a patient with lung adenocarcinoma who developed ED following sequential treatment with two third-generation EGFR-TKIs, aumolertinib and furmonertinib. Disease control was subsequently achieved after switching to the first-generation EGFR-TKI gefitinib. This case may serve as a reference for the management of severe cutaneous adverse reactions and for guiding subsequent individualized treatment strategies.

## 2 Case presentation

A 74-year-old female patient with no history of drug allergy presented in April 2025 with a paroxysmal headache persisting for more than two months. Imaging findings were indicative of centrally located lung cancer in the right lung with multiple brain metastases. Bronchoscopic biopsy was performed on April 8, 2025, and pathological examination confirmed lung adenocarcinoma on April 14, 2025. Whole-brain radiotherapy was administered on the same day (April 14, 2025). Genetic testing performed on April 17, 2025, indicated an *EGFR* exon 21 L858R variant.

Targeted therapy with aumolertinib at a dose of 110 mg once daily was initiated on April 21, 2025. On May 19, 2025, the patient developed erythematous skin rash with pruritus, which was not treated at that time. By May 25, 2025, the rash had worsened and was accompanied by fever. Treatment with loratadine at a local hospital demonstrated limited efficacy, and the patient was subsequently readmitted to the department due to persistent progression of the rash. On admission, the vital signs were as follows: temperature 37.1 °C; pulse 84 beats/min; respiratory rate 18 breaths/min; blood pressure 102/54 mmHg. Physical examination revealed generalized scattered erythematous rash and edema involving the face and extremities, with no other significant findings. The Karnofsky Performance Status (KPS) score was 50, and the Nutritional Risk Screening 2002 score was 3.

Laboratory results obtained on May 28, 2025 were as follows: white blood cell count 15.97×10^9^/L; neutrophil percentage 65.1%; eosinophil percentage 12.0%; absolute eosinophil count 1.92×10^9^/L; high-sensitivity C-reactive protein 53.10 mg/L; procalcitonin 0.231 ng/ml. The highest temperature recorded during hospitalization was 38.6 °C, and the patient became afebrile on June 2, 2025. Only aumolertinib had been administered during the month preceding the onset of the rash, and no history of drug allergy was reported. A clinical diagnosis of aumolertinib-induced ED was established, and the drug was discontinued immediately. The patient was treated with loratadine and thalidomide for anti-allergic therapy, piperacillin–tazobactam for anti-infection therapy, dexamethasone 5 mg daily beginning on June 3, 2025, and methylprednisolone 40 mg q12h starting on June 5, 2025, followed by doxycycline. Generalized skin desquamation developed on June 7, 2025 (**Figures 1A–C**), after which the rash gradually subsided. On June 8, 2025, the methylprednisolone dose was tapered to 40 mg daily. Skin desquamation indicated substantial improvement after two weeks (**Figures 2A, B**).

**Figure 1:**
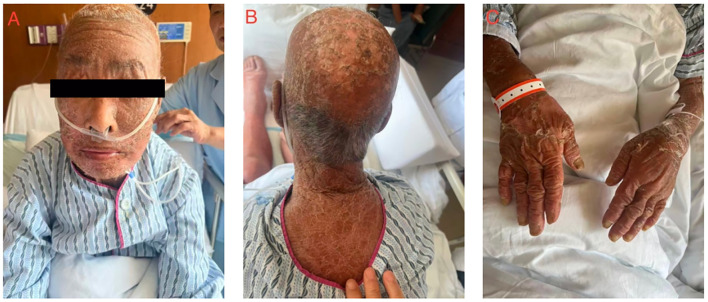
Clinical images presenting skin desquamation involving the head, face, and extremities following treatment with aumolertinib **(A–C)**.

**Figure 2:**
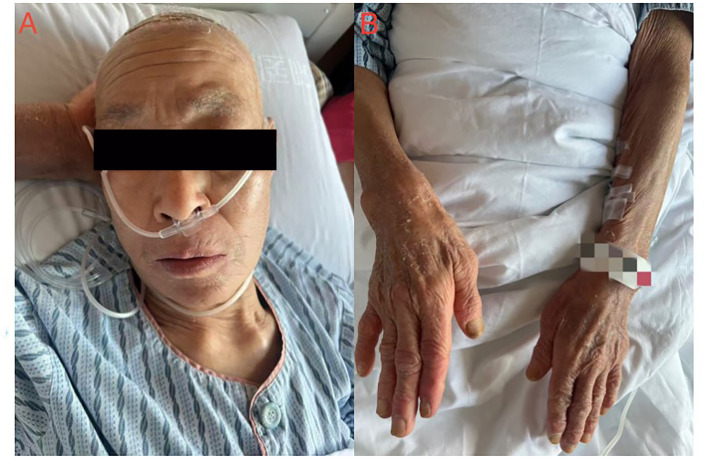
Clinical images presenting complete resolution of skin desquamation after drug withdrawal and symptomatic treatment **(A, B)**.

Treatment with furmonertinib, 80 mg once daily, was initiated on July 14, 2025. Six days later, the patient developed recurrent generalized rash that rapidly progressed to ED. Furmonertinib was discontinued immediately, and the patient presented to the outpatient clinic. Following treatment with antihistamines and systemic corticosteroids, the cutaneous lesions gradually resolved. During regular outpatient follow-up, erythroderma resolved completely approximately 10 days after treatment discontinuation. After written informed consent had been obtained, treatment was switched to the first-generation EGFR-TKI gefitinib, 250 mg orally once daily, on August 10, 2025. This treatment was well tolerated, and no recurrence of ED was observed. Chest computed tomography conducted on November 2, 2025, demonstrated a slight reduction in the right upper lobe mass compared with August 10, 2025 (**Figures 3A, B**). The therapeutic response was assessed as stable disease (SD), with disease control maintained for more than four months. The major clinical events are summarized in **Table 1**.

**Figure 3:**
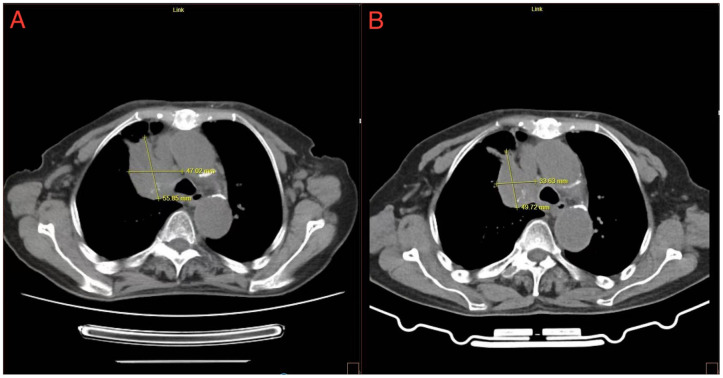
It showed shrinkage of lesions in the right upper lobe of the lung. **(A)** shows the non-contrast chest CT obtained on August 10, 2025, and **(B)** shows the non-contrast chest CT obtained on November 2, 2025 **(A, B)**.

**Table 1:** Timeline of the case.

Date	Clinical event	Management & outcomes
2025-04-14	Pathological examination of bronchoscopic puncture biopsy combined with imaging confirmed lung adenocarcinoma with brain metastases	Whole-brain radiotherapy was administered
2025-04-21	Genetic testing indicated EGFR exon21 L858R mutation	Targeted therapy with aumolertinib 110 mg once daily was initiated
2025-05-19	The patient developed erythematous rash with pruritus	No intervention was given
2025-05-25	The rash of the patient aggravated accompanied by fever	Loratadine treatment presented poor efficacy
2025-05-28	The patient was admitted to our department due to generalized rash	Aumolertinib was discontinued; anti-allergic, anti-infective and hormonal therapies were administered
2025-06-07	The patient developed generalized skin desquamation	Tapering of glucocorticoids
2025-07-14	The condition of the patient improved	Targeted therapy with furmonertinib 80 mg once daily was initiated
2025-07-20	Skin desquamation recurred	Furmonertinib was permanently discontinued
2025-08-10	The condition of the patient improved and chemotherapy was declined	Targeted therapy with gefitinib 250 mg once daily was initiated
2025-11-02	Chest CT presented a slight reduction of the mass in the right upper lobe compared with the previous scan (2025-08-10)	Efficacy was assessed as SD with shrinkage; disease control has been maintained for more than 4 months

## 3 Discussion

### 3.1 Mechanistic analysis of ED induced by aumolertinib and furmonertinib

Aumolertinib and furmonertinib are third-generation EGFR-TKIs that are clinically indicated for the treatment of NSCLC harboring *EGFR* sensitizing variants or T790M resistance variants (10). In clinical studies, both agents have generally demonstrated favorable tolerability. Cutaneous adverse reactions have been reported in approximately 21.8% of patients receiving aumolertinib, most often as mild rash; severe cutaneous adverse reactions, including Stevens–Johnson syndrome, appear to be rare. To the best of current knowledge, ED associated with aumolertinib has not been previously reported (11–13). Furmonertinib was likewise structurally optimized to reduce off-target toxicity, and severe cutaneous adverse reactions have not been reported in the published literature to date (14).

In the present case, aumolertinib was selected as the first-line treatment because of its demonstrated intracranial activity in patients with brain metastases (15). During treatment, the patient developed ED, which resolved following discontinuation of aumolertinib and appropriate supportive management. As the patient declined chemotherapy, alternative treatment options were considered. The patient’s KPS score was 50, indicating severe functional impairment. Although supportive care resulted in modest clinical improvement, her performance status remained inadequate for intensive chemotherapy. Standard platinum−doublet chemotherapy is generally associated with limited tolerability and modest clinical benefit in patients with poor performance status. Consequently, treatment with furmonertinib was initiated after careful consideration of the potential benefits and risks, including the possibility of recurrent cutaneous toxicity. At that time, the structural differences between aumolertinib and furmonertinib were considered to indicate a relatively low likelihood of cross-hypersensitivity. However, ED recurred shortly after furmonertinib initiation and resolved promptly after drug discontinuation. The recurrence of ED following sequential exposure to two structurally related third-generation EGFR-TKIs raises the possibility of cross-hypersensitivity within this drug class. Treatment was subsequently switched to the first-generation EGFR-TKI gefitinib, which was well tolerated, with no recurrence of ED during follow-up.

The observed clinical course—ED occurring during treatment with two different third-generation EGFR-TKIs but not after switching to the first-generation agent gefitinib—supports the hypothesis that the adverse reaction may have been mediated by an immunologic hypersensitivity mechanism rather than by non-specific pharmacologic toxicity. From a medicinal chemistry perspective, first- and third-generation EGFR-TKIs differ substantially in their chemical structures and mechanisms of target binding. First-generation EGFR-TKIs, such as gefitinib, possess a quinazoline core and bind reversibly to EGFR. In contrast, third-generation EGFR-TKIs share a pyrimidine core and an acrylamide moiety that enables irreversible covalent binding to cysteine 797 (Cys797) within the EGFR kinase domain. These conserved structural features, or the resulting drug–protein complexes, may function as common antigenic capable of eliciting T-cell-mediated delayed hypersensitivity reactions. Conversely, the distinct quinazoline scaffold and reversible binding characteristics of gefitinib may account for the absence of recurrent cutaneous hypersensitivity following the therapeutic switch.

This case highlights the importance of close monitoring for severe cutaneous adverse reactions during treatment with third-generation EGFR-TKIs. In patients who develop ED during treatment with a third-generation EGFR-TKI, caution should be exercised when considering a switch to another agent within the same generation because cross-hypersensitivity may occur. In contrast, switching to an EGFR-TKI with a different chemical scaffold, such as a first-generation agent, may represent a feasible therapeutic strategy in carefully selected patients.

### 3.2 Adverse reaction assessment

No history of ED was reported in this patient prior to the administration of aumolertinib, and no other medications were used during targeted therapy. These findings suggest a clear temporal association between aumolertinib and the onset of ED. According to the Naranjo scale, the total score for aumolertinib was 6, indicating a probable causal relationship between the drug and the adverse reaction. The patient’s symptoms gradually improved following drug discontinuation and supportive treatment. Recurrence of ED after subsequent administration of furmonertinib further supported a close association between third-generation EGFR-TKIs and the development of ED.

### 3.3 Diagnosis of ED

The onset of ED is often insidious and progressive; however, drug-induced cases may develop rapidly. Prodromal manifestations are generally nonspecific and may include pruritus, erythema, and mild desquamation, making early clinical recognition challenging. The initial diagnosis of ED primarily relies on clinical observation. A detailed medical history and comprehensive physical examination are essential for establishing the diagnosis (16). Currently, laboratory findings in ED also lack specificity. Most patients exhibit elevated erythrocyte sedimentation rate and C-reactive protein levels, and some may present with mild anemia, hypoproteinemia, neutrophilia, and eosinophilia. The combination of laboratory investigations and histopathological examination of biopsy specimens can further support and confirm the diagnosis (3).

### 3.4 Management of ED

The primary treatment for ED includes systemic glucocorticoids, combined with antihistamines and topical corticosteroids. High-dose intravenous immunoglobulin may be administered when necessary. In cases of secondary infection, appropriate antibiotic therapy should be initiated (3). In the present case, systemic glucocorticoids, including methylprednisolone and dexamethasone, constituted the principal anti-inflammatory and immunosuppressive treatment. Doxycycline was subsequently administered because of fever, elevated inflammatory markers, and extensive disruption of the skin barrier, which increased the risk of secondary bacterial infection. ED can lead to skin barrier dysfunction and life-threatening systemic complications, representing a potentially fatal condition (7).

Once ED is suspected, prompt intervention, accurate diagnosis, identification of the underlying cause, and appropriate treatment are required to improve patient prognosis (3).

### 3.5 Prevention strategies for ED caused by EGFR-TKIs

The early clinical manifestations of EGFR-TKI-associated ED lack specificity and are easily misdiagnosed as common allergic reactions or mild drug eruptions, potentially delaying timely intervention and leading to severe cutaneous injury. From a prognostic perspective, drug-induced ED can often be rapidly alleviated by prompt discontinuation of the causative agent and timely treatment (16).

Currently, no definitive preventive strategy or prophylactic medication is available in clinical practice to completely prevent EGFR-TKI-induced ED. During targeted therapy, clinicians should remain vigilant and adhere to the principles of dynamic monitoring, early detection, and timely intervention to reduce the risk of severe cutaneous adverse reactions and associated mortality.

## 4 Strengths and limitations

This case describes a rare occurrence of ED induced by EGFR-TKIs. Complete clinical data are presented for a patient with *EGFR*-variant lung adenocarcinoma who developed ED during treatment with third-generation EGFR-TKIs and achieved disease control after switching to a first-generation EGFR-TKI. This case may serve as a reference for treatment selection in patients who experience similar severe cutaneous adverse reactions.

Despite the comprehensive clinical documentation and a clear temporal relationship between drug exposure and adverse events, this report is limited by its single-case design. Further studies with larger sample sizes are needed to verify potential differences in cutaneous toxicity among different generations of EGFR-TKIs and to evaluate the safety and efficacy of switching between agents.

## 5 Conclusion

In this case, ED developed during treatment with third−generation EGFR−TKIs but did not recur after the patient was switched to the first-generation EGFR-TKI gefitinib. This clinical course suggests that switching to a first-generation EGFR-TKI may be a feasible option for selected patients who develop ED during treatment with a third-generation agent. However, evidence from a single case is insufficient to support a general treatment recommendation, and this observation requires validation in larger clinical studies. In patients with brain metastases, the relatively lower intracranial efficacy of first−generation EGFR-TKIs remains an important consideration. Decisions regarding intergenerational switching should therefore be individualized, taking into account the risk of recurrent cutaneous toxicity, intracranial disease burden, and overall systemic disease control. Close dermatological monitoring and early recognition of severe cutaneous adverse reactions are essential throughout EGFR-TKI treatment to facilitate prompt intervention and optimize patient outcomes.

## 6 Patient perspective

The patient stated: “During treatment with oral aumolertinib, I initially considered the rash to be a mild allergic reaction, as it improved after taking anti-allergic medication. I did not expect it to later progress to ED. Throughout the treatment process, I maintained trust in my attending physician. Following treatment, the ED resolved completely. When a change in the treatment plan was recommended, I was concerned that targeted therapy might cause the same severe skin reaction again; however, I was more worried about the adverse effects associated with chemotherapy. In addition, because the ED had previously resolved completely, I ultimately decided to continue oral targeted therapy.”

## Data Availability

The original contributions presented in the study are included in the article/supplementary material. Further inquiries can be directed to the corresponding authors.
